# Dietary diversity and associated factors among preschool children in selected kindergarten school of Horo Guduru Wollega Zone, Oromia Region, Ethiopia

**DOI:** 10.1186/s40795-022-00569-w

**Published:** 2022-07-29

**Authors:** Ebisa Olika Keyata, Abebe Daselegn, Alemayehu Oljira

**Affiliations:** 1 Department of Food Science and Nutrition, Faculty of Agriculture, Wollega University, P.O. Box 38, Shambu, Ethiopia; 2 Department of Chemical Engineering, Faculty of Technology, Wollega University, Shambu, Ethiopia; 3 Department of Agricultural Economics, Jimma University College of Agriculture and Veterinary Medicine, P.O. Box: 307, Jimma, Ethiopia

**Keywords:** Associated factors, Dietary diversity, Kindergarten school, Preschool children

## Abstract

**Background:**

Preschool children are the most vulnerable group because of their high nutritional needs for growth and development. The study assessed dietary diversity scores and associated factors among preschool children in selected kindergarten schools of Horo Guduru Wollega Zone, Western Ethiopia.

**Methods:**

The institutional-based cross-sectional study design was conducted on a total 440 of preschool children. A semi-structured questionnaire was used to collect information on the dietary diversity score of preschool children using a 24 h dietary recall method. Binary logistic regression was used to identify variables associated with dietary diversity scores of preschool children.

**Results:**

The result showed that the majority (87.3%) of preschool children in the selected kindergarten school practiced a low dietary diversity score (less than four food groups). The result obtained from multivariate logistic regression analysis indicated that the age of preschool children [AOR 9.58(2.26–40.60)], sex of child [AOR 3.21(1.71–5.99)], and work of mother [AOR 7.49(2.33–24.07)] were significantly (*p* < 0.05 associated) with dietary diversity of children.

**Conclusions:**

The findings indicated that many preschool children in the study area did not get a minimum dietary diversity score. Therefore, health extension workers must organize community-based behavior change nutritional education for mothers or caregivers to create awareness of preschool child dietary diversity practices.

## Introduction

Dietary diversity is described as the consumption of various food types such as vegetables, fruits, grains, tuber, meat, and dairy product during a given time. It reflects the idea that diversifying one's diet by including a wider range of foods and food groups helps ensure optimal nutritional intake [1]. Institutional-based diet can be defined as entities that provide meals at institutions, including schools, colleges and universities, hospitals, correctional facilities, public and private cafeterias, nursing homes, and day-care and senior centres [2]. Among the indicated institution, nutrition for school-aged children is important for vulnerable groups, particularly preschool children, because it substantially impacts their health, cognition development, and educational achievement [3].

Appropriate and adequate feeding practice of various food groups for children and infants was fundamental for optimal child growth and development [4]. However, low dietary diversity may be the major cause of malnutrition [5]; mainly, it is a crucial problem that leads to chronic and non-communicable diseases in the future life [6] and is boldly perceived among the rural community and poor populations [7].

In the world, 10.9 million (60%) death occurred in under 5 years. Of those, 66% of deaths were due to poor feeding practices during the first 2 years of life [8, 9]. In developing countries, inappropriate dietary diversity is the major reason for childhood malnutrition which is strongly associated with morbidity and mortality [10, 11].

In Ethiopia, dietary diversity is a major challenge because preschool children consume a less diversified diet from the family dish, which is mainly cereal-based [12]. This poor dietary diversity feeding practice of preschool children resulted in a decline in education quality due to the student falling out of the classroom and discontinuing school [13].

Starchy-based staple diet with inadequate animal products, fresh fruits, and vegetables is highly practiced for infants and young children in Horo Guduru Wollega zone, Oromia, Ethiopia [14]. Ekesa et al. [15] showed that the main causes of poor dietary diversity could be resource limitation, information inadequacy, and lack of access to dietary diversity. Consequently, chronic undernutrition is very high among children who eat monotonous diets [16]. Different scholars indicated that numerous household socioeconomic status and cultural factors affect dietary diversity scores and feeding practices of children [17, 18, 19].

Families with greater incomes tend to have more diverse diets, and their children grow better for several reasons [20]. However, lower intakes of food sources have also been observed among children in low-income households. Additionally, culture, religion, and traditional knowledge affect food and nutrition security by shaping communities' diets and intra-household food distribution patterns, affecting dietary diversity scores and intake of nutrient-rich foods in children [21].

Appropriate and adequate feeding practices are a prerequisite for good nutritional status in any given time of human life [22]. However, a lack of dietary diversity can have negative consequences (impact on the age at any time) at any age, but is particularly critical for infants and young children, especially on their health, well-being, and development, mainly by reducing physical capacities as well as resistance to infectious diseases [8]. This might be due to inadequate consumption of fruit and vegetable, which is rich in essential micronutrients such as vitamin A, iron, and zinc. These deficiencies affect the survival, health, development, and well-being of those afflicted.

Studies in Ethiopia identified dietary diversity scores and their associated factors among children aged between 6 to 23 months [14, 23, 9, 24]. The authors mentioned above indicated that there was poor dietary diversity and their diets were mainly cereal and pulse-based diets. However, there is limited information regarding dietary diversity and its associated factors among preschool children aged between 4-and 7 years in Ethiopia and study areas in particular. Hence, the present study was designed to fill this gap by addressing dietary diversity scores and associated factors among preschool children aged between 4-and 7 years in the selected kindergarten school of Horo Guduru Wollega Zone, Oromia, Ethiopia.

## Methods

### Description of the study area

The study was conducted in Horo Guduru Wollega Zone, Oromia, Western Ethiopia. The capital town of the zone, Shambu, is located 314 km from Addis Ababa in the Western part of Ethiopia. The zone comprises eleven rural districts. According to the report (CSA, 2011), the Horro Guduru Wollega zone covers a total land area of 8,097 km2; a total population of 641,575, of which 50.09% are male, and 49.91% are female. This study was conducted in Shambu town (Abishe Gerba, Catholic & Mati Boru Kindergarten School) and Fincha town (Fayissa & Horo Guduru College Kindergarten School), located to the west of the capital city of Ethiopia (Addis Ababa). Five kindergarten schools were selected based on the high number of preschool children in the Horo Guduru Wollega zone.

### Study design

An institution-based cross-sectional survey design was conducted to investigate the dietary diversity and its associated factors of preschool children.

### Study population

All preschool children aged 4 to 7 years with their mothers/ caregivers who are randomly selected from the selected kindergarten and who met the inclusion criteria were the study population. Children aged 4 to 7 years who did not come to school and were sick during data collection were excluded from the study.

### Sample size determination

A single population proportions formula was used to determine the sample size of the study population using the assumption that 46% of the preschool children were low dietary diversity category in Matungu, Butere-Mumias region of western Kenya [25]. A Zα/2 had a 95% confidence level of 1.96, a margin of error of 0.05, a non-response of 10%, and a design effect of 1.5 to correct the sampling error. Accordingly, the calculated sample was 440 using the Stat CALC application of EpiinfoTM 7.0.8.3.

### Sampling procedure

In this study, two sampling techniques were used to select study participants. In the first stage, the purposive sampling technique was used to select a kindergarten school in the Horo Guduru Wollega zone based on different preschool children in the town. Accordingly, three kindergarten schools were selected in Shambu town (Abishe Gerba, Catholic & Mati Boru) and two kindergarten schools in Fincha town (Fayissa & Horo Guduru College Kindergarten School). In the second stage, a simple random sampling technique was used to select the target group from the selected kindergarten school based on the proportion allocation ratio proposed in each school.

### Data collection tool and procedures

Data were collected using an interviewer-administered questionnaire from preschool children's mothers/ caregivers by allowing them freely to recall the type of food items they fed to their child or children within the last 24 h. The questionnaire used in this study was first prepared in the English language and translated into the Afan Oromo. The first part of the questionnaire contains items used to collect socio-demographic information adopted from the Ethiopian Demographic and Health Survey questionnaire after contextual modifications were done. Dietary diversity was assessed by asking the mothers/ caregivers whether their preschool children obtained food from the seven food groups in the last 24 h. Dietary Diversity Score (DDS) ranges from zero (0) to seven (7) which is computed by summing the number of unique food groups the child received in the last 24 h.

### Data quality control

Two preschool teachers and one preschool director in each kindergarten school were selected, and then training was given to them. Overall, data were collected by ten preschool teachers and five school directors, and the researchers. At the end of each day, the principal investigator checked the completeness of the questionnaires. Before data collection, the questionnaire was pre-tested on 5% of the sample size out of sampled using pilot interviews with mothers/caregivers of preschool children.

### Study variable

Child dietary diversity score (DDS) was considered a dependent variable. In contrast, socio-demographic variables including age, sex, a cycle of the child, ethnicity, religion, education of father, work of father and mother, family size, and obtained food were recorded as independent variables.

### Children's dietary diversity

Dietary diversity was determined based on 24-h dietary recall methods. The mothers of preschool children have requested to state what their children consumed the previous day. Dietary diversity was then computed based on seven food groups as recommended by WHO [20], which comprise cereal, roots, and tubers; legumes and nuts; dairy products; flesh foods (meat, fish, poultry, and organ meats); eggs; vitamin-A rich fruits and vegetables; Fruits (mango, papaya, orange, avocado, banana, pineapple) and Vegetables (leaf, leaf of pumpkin, cabbage, lettuce) and other fruits and vegetables such as kale, Kosta, etc. Consumption of food from each food group was sufficient to count except if a food item was only used as a condiment. However, oils and fats were not considered and calculated for dietary diversity score; these foods don't add nutritional quality to the diets.

The dietary diversity score was calculated for each preschool child during the previous 24 h to categorize the children's dietary diversity as high DD (> 4 food groups) or low DD (< 4 food groups) from seven food groups. Then, the outcome variable was coded as a high DD > 4 food groups as "1" and low DD < 4 food groups as "0" for logistic regression analysis.

### Data processing and analysis

Data obtained were coded, entered, and analyzed using SPSS for windows version 20.0. Frequencies and percentages were conducted to summarize the descriptive part of preschool children's socio-demographic characteristics and consumption of food groups. A binary/multivariate logistic regression was done to identify factors associated with dietary diversity scores. The dependent variable was coded as '1' for those who had consumed four or more foods and '0' for less than four food groups during the previous 24 h. In the beginning, the association between each independent and dependent variable was examined using bivariate logistic regression; then, variables that showed significant associations based on the assumptions were considered for multivariate logistic regression. Finally, significant variables were identified in multivariate logistic regression at a p-value < 0.05.

## Results

### Socio-demographic characteristics of preschool children

The result showed that less than half of the preschool children aged 5 years were enrolled in kindergarten. The gender representation for the 4 to 7-year-old children was 191 (43.4%) for boys and 249(56.6%) for girls. The majority, 234(53.2%) of preschool children, exist in the first cycle. Most of the respondents were Oromo by ethnicity 426 (96.8%) and protestant by religion 249 (56.6%). The parents' education level showed that the majority of the father attended school and were employed with a diploma and above 231(52.5%). Regarding the occupational status of caregivers, more than half of 275(62.5%) of the father employed in the government office, while 181(41.1%) of the mothers were government employees. The majority, 294 (66.8%) of household sizes, were up to five people per household, including children (Table 1).

**Table 1:** Socio-demographic characteristics of preschool children in the study area

Variables	Category	Frequency (*n* = 440)	Percent
Age of preschool children (years):	4	40	9.1
	5	191	43.4
	6	152	34.5
	7	57	13.0
Sex of child	Boy	191	43.4
	Girl	249	56.6
Cycle of child	First cycle	234	53.2
	Second cycle	206	46.8
Ethnicity	Oromo	426	96.8
	Amhara	14	3.2
Religion	Orthodox	150	34.1
	Catholic/protestant	249	56.6
	Muslim	41	9.3
Educational status of Father	Illiterate	33	7.5
	Primary School	97	22.0
	Secondary School	79	18.0
	Diploma and above	231	52.5
Occupational status father	Employed	275	62.5
	Farmer	52	11.8
	Self-business	113	25.7
Occupation status of the mother	Employed	181	41.1
	Housewife	165	37.5
	Self-business	94	21.4
Household size	1 up to 5	294	66.8
	Greater than 5	146	33.2
Food obtained	Buying	325	73.9
	Farming	115	26.1

### Dietary diversity score of preschool children

The mean (± SD) intake of dietary diversity score of preschool children aged four to five years old was 2.67 (± 0.80). The results showed that a high proportion of 384 (87.30%) of the study participants were categorized in the lowest dietary diversity score (DDS), while 56(12.7%) were in the high dietary diversity score. The majority of the preschool children had consumed all starchy staple foods (cereals, roots, and tubers (100%) and pulses (legumes and nuts) (70.5%) in the previous 24 h. A quarter of preschool children aged 4–7 years had consumed eggs and Vitamin A-rich fruits and vegetables. The least consumed food group was other fruits and vegetables (1.4%) (Table 2).

**Table 2:** Consumption of food groups by preschool children in the study area

Variables	Category	Frequency (*n* = 440)	Percent
Cereals, roots, and tubers	No	0	0
	Yes	440	100.0
Legumes and nuts	No	130	29.5
	Yes	310	70.5
Dairy products	No	398	90.5
	Yes	42	9.5
Flesh foods	No	289	65.7
	Yes	151	34.3
Eggs	No	326	74.1
	Yes	114	25.9
Vitamin A-rich fruits and vegetables	No	326	74.1
	Yes	114	25.9
Other fruits and vegetables	No	434	98.6
	Yes	6	1.4
Overall dietary diversity scores	High	56	12.7
	Low	384	87.3

### Factors that associated with Dietary Diversity of preschool children

In bivariate logistic regression analysis, children’s age, sex of a child, a cycle of a child, and the work of father and mother were the candidate variables for multi-variables analysis. Whereas in multivariable logistic regression analysis, age of preschool children [AOR 9.58(2.26–40.60)], sex of child [AOR 3.21(1.71–5.99)], and work of mother [AOR 7.49(2.33–24.07)] were significantly associated with dietary diversity of children as indicated in Table 3.

**Table 3:** Bivariate and multivariate logistic regression analysis of factors associated with minimum dietary diversity among preschool children aged 4–7 years in the study area

**Variables**	**Category**	**Dietary Diversity Score**		**Crude odds ratio (COR) 95% CI**	**Crude odds ratio (AOR) 95% CI**	***P*-value**
		**Low**	**High**			
Age of Preschool children (years):	4	32(8.30)	8(14.30)	1	1	0.000^*^
	5	174(45.30)	17(30.4)	11.60(2.47,54.35^)*^	9.58(2.26,40.60)	0.002^*^
	6	130(33.9)	22(39.3)	2.45(0.75,8.01)^*^	1.62(0.54,4.91)	0.39
	7	48(12.5)	9(16.1)	1.38(0.50,3.85)	0.73(0.25,2.16)	0.57
Sex of child	Boy	181(47.1)	10(17.9)	1	1	
	Girl	203(52.9)	46(82.1)	3.28(1.66,6.48)^*^	3.21(1.71,5.99)	0.000^*^
Cycle of child	first cycle	212(55.2)	22(39.3)	1	1	
	second cycle	172(44.9)	34(60.7)	0.43(0.18,1.02^)*^	0.45(0.20,1.03)	0.058
Ethnicity	Oromo	373(97.1)	53(94.6)	1	-	-
	Amhara	11(2.9)	3(5.4)	0.00 (0.00)		
Religion	Muslim	30(7.8)	11(32.1)	1	-	-
	Orthodox	123(32)	27(48.20)	0.00(0.00)		
	Protestant	231(60.2)	18(32.10)	1.84(0.89,3.81)		
Education of father	Illiterate	30(7.8)	3(5.4)	1	-	-
	Primary School	86(22.4)	11(19.6)	0.97(0.23,4.04)		
	secondary School	70(18.2)	9(16.1)	0.44(0.17,1.10)		
	diploma and above	196(51.6)	33(58.90)	0.45(0.17,1.18)		
Work of father	Employed	231(60.2)	44(78.6)	1	1	0.40^*^
	Farmer	52(13.5)	0(0)	0.38(0.16,0.88)	0.63(0.31,1.29)	0.21
	Self-business	101(26.3)	12(21.4)	0.30(0.97,0.95)	0.59(0.21,1.64)	0.31
Work of mother	Employed	148(38.5)	33(58.9)	1	1	0.003^*^
	Housewife	145(37.8)	20(35.7)	4.85(1.39,16.79)^*^	4.19(1.30,13.53)	0.02^*^
	Self-business	91(23.7)	3(5.4)	7.95(2.37,26.70^)*^	7.49(2.33,24.07)	0.001^*^
Household size	1 up to 5	263(68.5)	31(55.4)	1	-	-
	greater than 5	121(31.5)	25(44.6)	1.87(0.81,4.29)		
Food obtained	Buying	280(72.9)	45(80.40)	1	-	-
	Farming	`104(27.1)	11(19.6)	0.59(0.29,1.20)		

*COR* Crude odds ratio, *AOR* Adjusted odds ratio

*: indicated signifcant variable at *p*-value <0.05 in adjusted odds ratio for multivariate

## Discussion

In this study, all preschool children consumed cereal-based foods made from teff, maize, wheat, oat, and barley, with relatively low nutrient density. The findings were with the Horo district's findings, showing that 97.7% of children consumed food from cereal, roots, and tubers [14]. However, this study contradicted Ethiopia's Demographic health survey [26], children consumed foods made from grains (66%) [27]. This might be due to the food consumption of children in the rural area being monotype, cheap and affordable.

The study also revealed that most preschool children aged 4–7 years had consumed legumes and nuts during the past 24 h. The obtained results were lower than the study conducted in the Horo district that showed 84.6% of children had the habit of eating daily plant protein such as bean, pea, and lentils prepared stew consumed with injera staple diet for the majority of Ethiopians [14].

The findings also depicted that most preschool school children aged 4–7 didn't consume any cheese, yoghurt, milk, or other dairy products in the past 24 h. This finding was similar to a study in the Amhara region in Finote Selam town, where 89.60% of preschool children didn't consume milk and milk products [28].

Less than half of preschool children considered in this study had consumed fresh food (meat, fish, and poultry) in the last 24 h. The findings were more than two times higher than the study conducted in Shashemene [29] (14%). This might be due to the improvement of knowledge and perception of mothers/caregivers during preparing food for preschool children (4 to 7 years) who can digest fatty foods compared to children aged 6 to 23 months.

A quarter of preschool children aged 4–7 years had consumed eggs. This result is lower than the study conducted in both Shashemene (40.1%) [29] and Horo district (30.4%) [14] for children between 6 to 23 months but higher than the study conducted in India [30], which showed that less than 8% of all children aged 6—23 months had consumed eggs. The study revealed that a quarter of preschool children had consumed Vitamin A-rich fruits and vegetables. This result is in line with the study conducted in Shashemene woreda [29]. This finding is also comparable with other studies conducted in Kenya [31, 32].

The overall results regarding the dietary diversity score of preschool children showed that most preschool children didn't achieve a minimum dietary diversity score. However, less than a quarter of preschool children met the minimum dietary diversity score requirements in the last 24 h. The result found was lower than the study conducted in the Diredawa city, Ethiopia (24.4%) [33] and Tanzania (38%) [11]. However, the obtained result was higher when compared to the Ethiopia Demographic & Health Survey [26], in which 5% of children had minimum dietary diversity [26].

Preschool children aged four and five years were more likely to initiate dietary diversity than children who were six and seven years of age. This might be because many children aged four and five years may give special consideration to eating different food groups from their mothers/caregivers.

The findings obtained in this study revealed that girl children were 4 times more likely to meet minimum dietary diversity compared to boy children. This might be because they gave more attention to feeding their girls various food items than a male child. The results contrast with the study conducted in Diredawa, Ethiopia, which reported that male children were 3 times more likely to meet minimum dietary diversity than female children because of traditional influence on male sex preference [33].

The findings also showed that mothers' work had a statistically significant (*p* < 0.05) association with children's dietary diversity. Children born from mothers who had self-business and housewives were 7.49 and 4.19 times more likely to practice the recommended dietary diversity than those born from their mothers employed in governmental organizations. This might be due to a lack of time to give different food groups for their child when related to the mother housewife.

## Conclusion

In this study, dietary diversity score and associated factors among preschool children in selected kindergarten school of Horo Guduru Wollega Zone, Oromia Region, Ethiopia was reported. The findings showed that a high percentage of preschool children aged 4–7 years old didn’t get minimum dietary diversity as recommended by World Health Organization. Besides this, the consumption of animal-source foods such as dairy products, MPF (meat, poultry, and fish), eggs, vitamin A-rich fruit &vegetables, and other fruit and vegetable was very poor among the surveyed preschool children in the study area. The result also clearly indicated that the age of the child, sex of the child, and work of the mother are significantly associated with dietary diversity practices. Therefore, health extension workers need to organize community-based behavior change nutritional education for mothers or caregivers to create awareness of preschool child feeding practices. Agricultural extension workers should also be aware and train farmers to boost the production of animal source foods, Vitamin A-rich fruits and vegetables, and other fruits and vegetables through rearing small animals and irrigation activities to meet the minimum dietary diversity of children.

## Data Availability

All data collected from the respondents are included within the manuscript.
